# Effect of calcium electroporation on tumour vasculature

**DOI:** 10.1038/s41598-018-27728-z

**Published:** 2018-06-20

**Authors:** Barbara Staresinic, Tanja Jesenko, Urska Kamensek, Stine Krog Frandsen, Gregor Sersa, Julie Gehl, Maja Cemazar

**Affiliations:** 1 Department of Experimental Oncology, Institute of Oncology Ljubljana, Zaloska cesta 2, Ljubljana, Slovenia; 2 University of Ljubljana, Faculty of Medicine, Vrazov Trg 2, Ljubljana, Slovenia; 3 Center for Experimental Drug and Gene Electrotransfer (C*EDGE), Department of Clinical Oncology and Palliative Care, Zealand University Hospital, Roskilde, Denmark; 4 University of Ljubljana, Faculty of Health Sciences, Zdravstvena pot 5, Ljubljana, Slovenia; 5 Department of Clinical Medicine, Faculty of Health and Medical Sciences, University of Copenhagen, Copenhagen, Denmark; 6 University of Primorska, Faculty of Health Sciences, Polje 42, Izola, Slovenia

## Abstract

Calcium electroporation (CaEP) is a novel anti-tumour treatment that induces cell death by internalization of large quantities of calcium. The anti-tumour effectiveness of CaEP has been demonstrated *in vitro*, *in vivo*, and in preliminary clinical trials; however, its effects on the vasculature have not been previously investigated. Using a dorsal window chamber tumour model, we observed that CaEP affected to the same degree normal and tumour blood vessels *in vivo*, as it disrupted the vessels and caused tumour eradication by necrosis. In all cases, the effect was more pronounced in small vessels, similar to electrochemotherapy (ECT) with bleomycin. *In vitro* studies in four different cell lines (the B16F1 melanoma, HUVEC endothelial, FADU squamous cell carcinoma, and CHO cell lines) confirmed that CaEP causes necrosis associated with acute and severe ATP depletion, a picture different from bleomycin with electroporation. Furthermore, CaEP considerably inhibited cell migratory capabilities of endothelial cells and their potential to form capillary-like structures. The finding that CaEP has anti-vascular effects and inhibits cell migration capabilities may contribute to the explanation of the high efficacy observed in preclinical and clinical studies.

## Introduction

Electroporation is a physical method for permeabilization of cell membranes, allowing molecules and ions that are normally unable to cross the membrane because of their chemical and physical properties to enter the cell and reach their intracellular targets^1^. ECT combines electroporation and chemotherapeutic drugs to enhance local cytotoxicity and limit systemic toxicity^2^. The most commonly used drugs for ECT are bleomycin and cisplatin^3,4^.

Recently, a combination of calcium and electroporation has emerged as an anti-tumour treatment. Calcium is an important and ubiquitous second messenger involved in the regulation of a wide variety of cellular processes, including proliferation and cell death, and its cytosolic concentration is strongly maintained at low levels^5^. Excessive influx and uptake of calcium in cellular storages, such as in the endoplasmic reticulum and mitochondria, signifies cell stress and can lead to overload, which consequently causes cell death through mitochondrial dysfunction and subsequent energy production failure^6–9^. CaEP was initially investigated as a method to turn off transfected genes^10^ and was later investigated for its anti-cancer properties^11^.

A contributing mechanism of CaEP is ATP depletion, as the cells are exposed to a sudden loss of ATP likely due to increased consumption and impaired production of ATP. Other mechanisms may involve activation of lipases and proteases and generation of reactive oxygen species (ROS)^8,12,13^. In the first preclinical study, CaEP showed a decrease in viability and amount of ATP in 3 different cancer cell lines *in vitro*. In the same study, the effect of CaEP was tested *in vivo* on small-cell lung cancer, where complete necrosis was observed in 89% of tumours^11^. Further studies investigated different concentrations of calcium, and dose-dependent decreases in viability and intracellular ATP were observed^14,15^. The effect of CaEP was also tested in spheroids of normal cells and cancer cells, all of which responded with a similar extent of ATP depletion; however, the viability of normal fibroblast spheroids appeared to be less affected^16^. Recently, this was confirmed *in vivo*, where normal skin and muscle tissue treated with CaEP were less affected than tumour tissue^17^. However, normal cells in suspension treated with CaEP have shown a similar response to cancer cells, indicating that the toxicity of CaEP is different when cells are freely suspended^18^. The first clinical trial testing CaEP was recently completed and showed similar response rates as ECT^19^. Furthermore, a case report of a patient with a systemic immune response and remission of disseminated malignant melanoma after ECT with bleomycin and CaEP was published, showing that CaEP might not only be a local cancer treatment but can also induce a systemic response^20^.

ECT with bleomycin is already a well-researched and established treatment that has a blood flow modifying effect and vascular disrupting action, in addition to direct cytotoxic effects^21,22^, which may indeed be an important contributor to the high clinical efficacy observed. However, electroporation with calcium is a novel approach and its effects on the vasculature have not yet been examined. Hence, the aim of our study was to investigate the anti-vascular effects of CaEP, in comparison to ECT with bleomycin as a positive control.

## Results

### CaEP affects cell viability and causes ATP depletion

**Figure 1:**
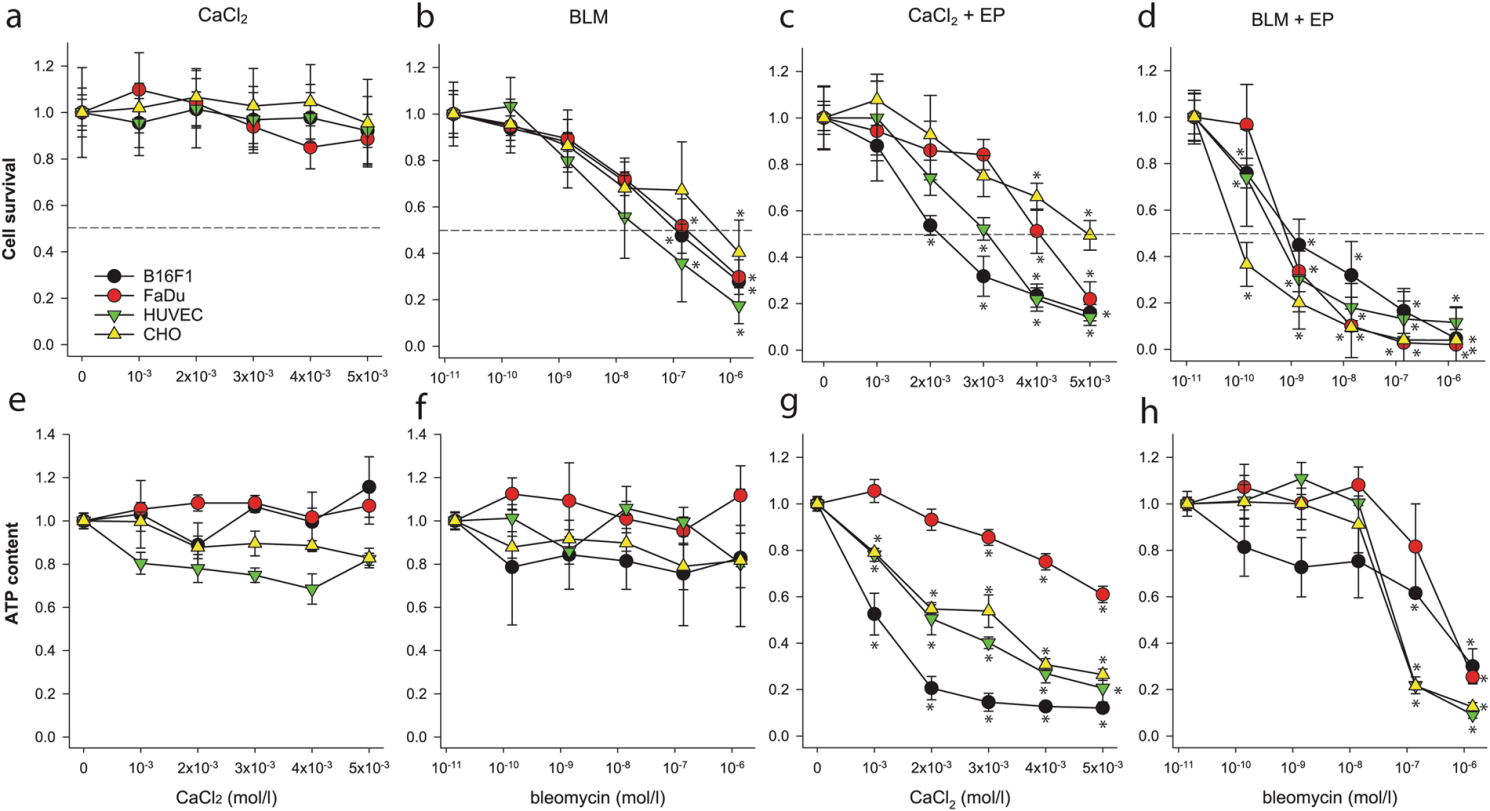
Cytotoxicity and ATP depletion after treatment with CaEP or ECT with bleomycin. (**a**–**d**) Relative survival of different normal and tumour cell lines 72 hours after treatment with increasing concentrations of calcium or bleomycin and in combination with electroporation (AM ± s.e.m.; n = 16 wells in 3 independent experiments). (**e**–**h**) The relative concentration of intracellular ATP content 1 hour after treatment with increasing concentrations of calcium or bleomycin and in combination with electroporation (AM ± s.e.m.; n = 4 wells in 3 independent experiments). *p < 0.05, statistical comparisons were performed by one-way ANOVA analysis and compared to control groups by Holm-Sidak testing. All data are normalized to the untreated or electroporated-only cells.

CaEP exhibited cytotoxic effects in a dose-dependent manner in all cell lines, but with differing sensitivities. IC_50_ concentrations of calcium and bleomycin (combined with electroporation) were calculated from survival curves for each cell line (Fig. 1a–d). Obtained IC_50_ values for calcium were 2.2 mM (B16F1, murine melanoma cells), 4 mM (FaDu, human pharynx squamous cell carcinoma), 3.1 mM (HUVEC, human umbilical vein endothelial cells) and 5 mM (CHO cells, Chinese hamster ovary cells). For bleomycin, IC_50_ concentrations were 0.87 nM (B16F1), 0.57 nM (FaDu), 0.39 nM (HUVEC) and 0.069 nM (CHO). The intracellular concentration of ATP after both therapies was also measured (Fig. 1e–h). A significant decline in ATP concentration after CaEP was detected in all cell lines, showing a trend similar to that observed for cell survival. At the IC_50_ calcium concentrations, in B16F1 cells, ATP levels dropped to 20–30%, in FaDu cells to 70–80%, in CHO cells to 20–30% and in HUVECs to 40–50%. The normal CHO cells were less sensitive to CaEP and they appeared to overcome ATP depletion, whereas, after ECT, they exhibited the highest sensitivity. B16F1 melanoma cells were the most sensitive to CaEP since there was the most significant drop in cell viability and intracellular ATP. ECT with bleomycin also resulted in a dose-dependent decline in cell viability, whereas ATP depletion was observed only at the highest doses of bleomycin, where most of the cells were dead. Treatment with calcium alone did not show any decrease in survival or ATP concentrations; however, treatment with bleomycin alone showed a significant decrease in survival, but not in ATP levels.

### CaEP induces cell death

**Figure 2:**
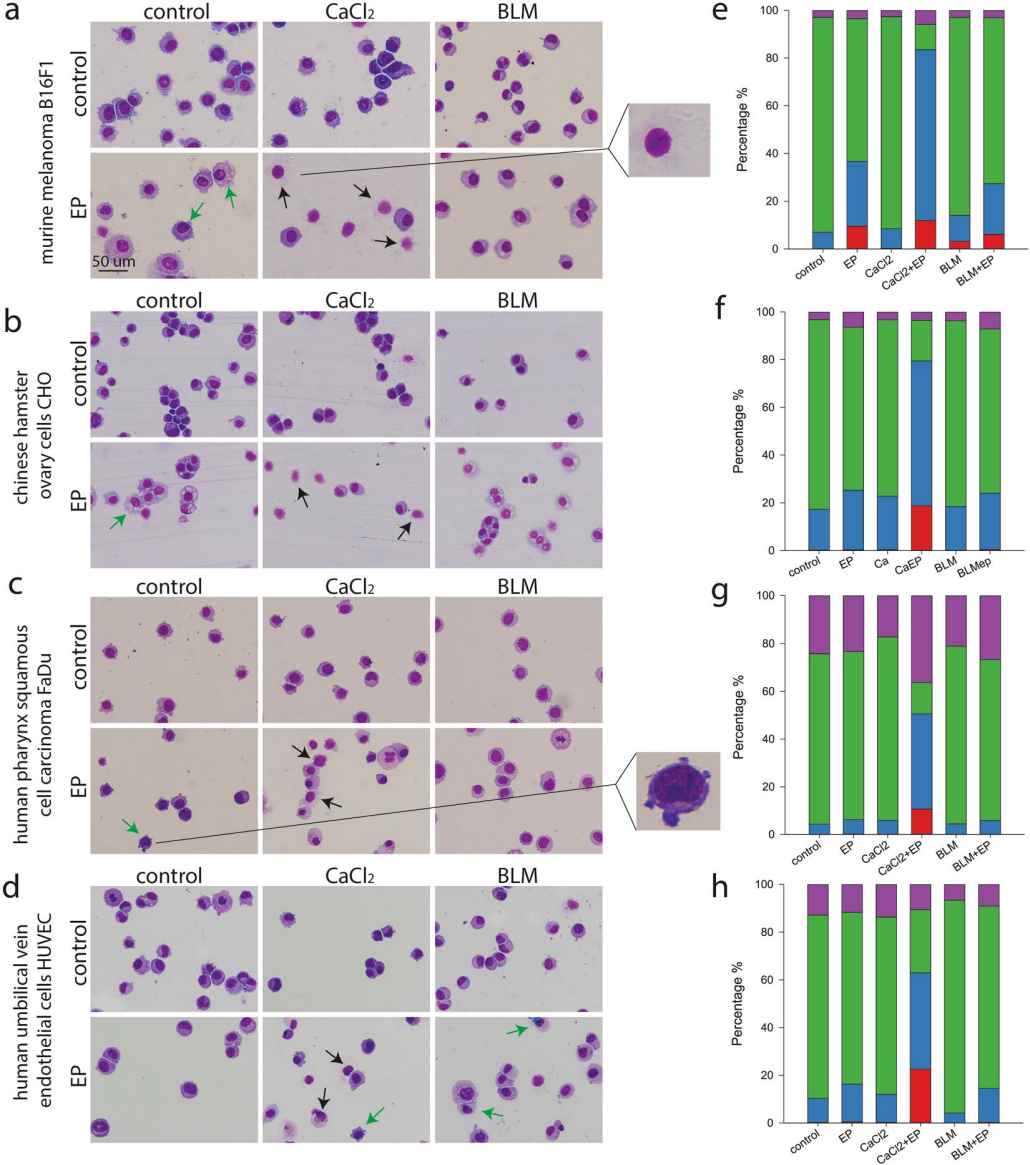
Cell morphology and type of cell death after CaEP or ECT with bleomycin (BLM). (**a**–**d**) Black arrows represent necrotic cells with ruptured membranes and cell lysis (example magnified on upper right). Green arrows indicate cells with early signs of apoptosis, such as vacuolisation of cytosol (example magnified on lower right). Morphological changes were assessed in at least two independent experiments with two parallels. (**e**–**h**) Percentage of viable cells (negative; green), apoptotic cells (Annexin V positive; purple), late apoptotic/necrotic cells (Annexin V + 7-AAD positive; blue) and necrotic cells (7-AAD positive; red) after different treatments is shown. Each graph corresponds to the cell line in the same row. Bars represent the average of the experiment with two parallels repeated twice.

The cell morphology after CaEP or ECT with bleomycin was observed after Giemsa staining (Fig. 2a–d). Almost no cell death was observed in control samples, whereas a major increase of cell death was observed after CaEP. These findings indicate that necrosis is the most likely cell death mechanism after CaEP, as previously seen *in vivo*^11,17^. To confirm observed cytotoxic effects and changed morphology, flow cytometric analysis of cells labelled with FITC Annexin V and 7-AAD was performed to determine cell death mechanism (Fig. 2e–h). After CaEP cells were predominately necrotic or in late necrotic/apoptotic stage. Rupture of cell membranes and cell lysis were the most visible features for cells dying by necrosis^23^. After EP alone and ECT with bleomycin, cell swelling was observed. In addition, after ECT some early signs of apoptosis were observed^24^, such as vacuolisation in the cytosol and nuclear condensation; however, this was not confirmed with flow cytometric measurements and could represent also other types of cell death (Fig. 2e–h).

### CaEP inhibits tube formation and cell migration of endothelial cells

**Figure 3:**
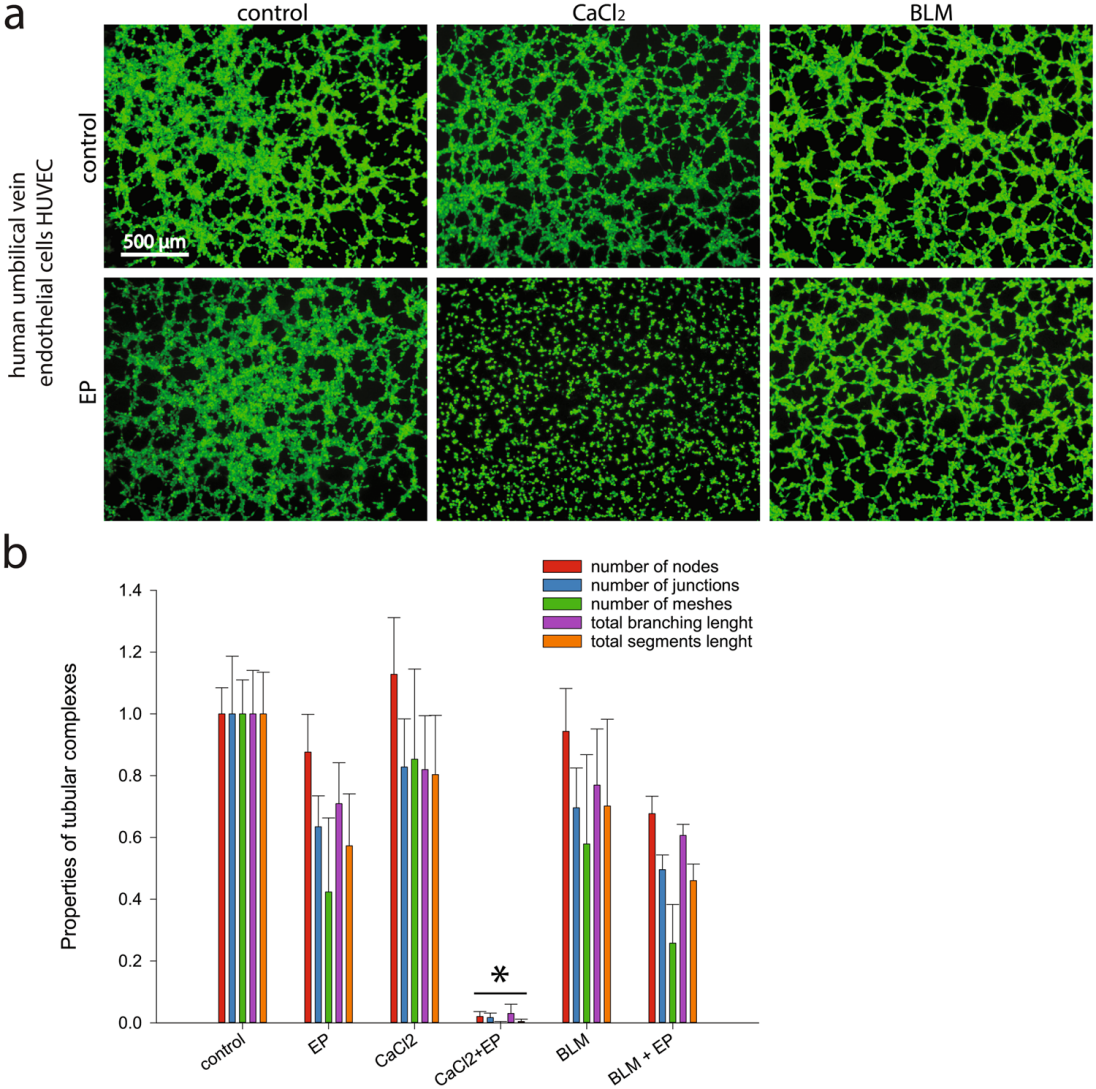
Reduced ability to form capillary-like structures of HUVEC endothelial cells after CaEP or ECT with bleomycin (BLM). (**a**) Original images of tubular complexes 4 hours after treatment with IC_50_ concentrations of calcium or bleomycin and in combination with electroporation. (**b**) Analysis of tubular complexes by quantification of different properties (number of nodes, junctions, meshes, total branching length and total segments length). All data were normalized to non-treated control cells. Comparisons between groups were carried out by Holm-Sidak testing following one-way ANOVA, an asterisk indicates the statistically significant difference (*p < 0.05). Results represent at least two independent experiments, n = 4 or more wells in each experiment. Bars represent AM ± s.e.m.

**Figure 4:**
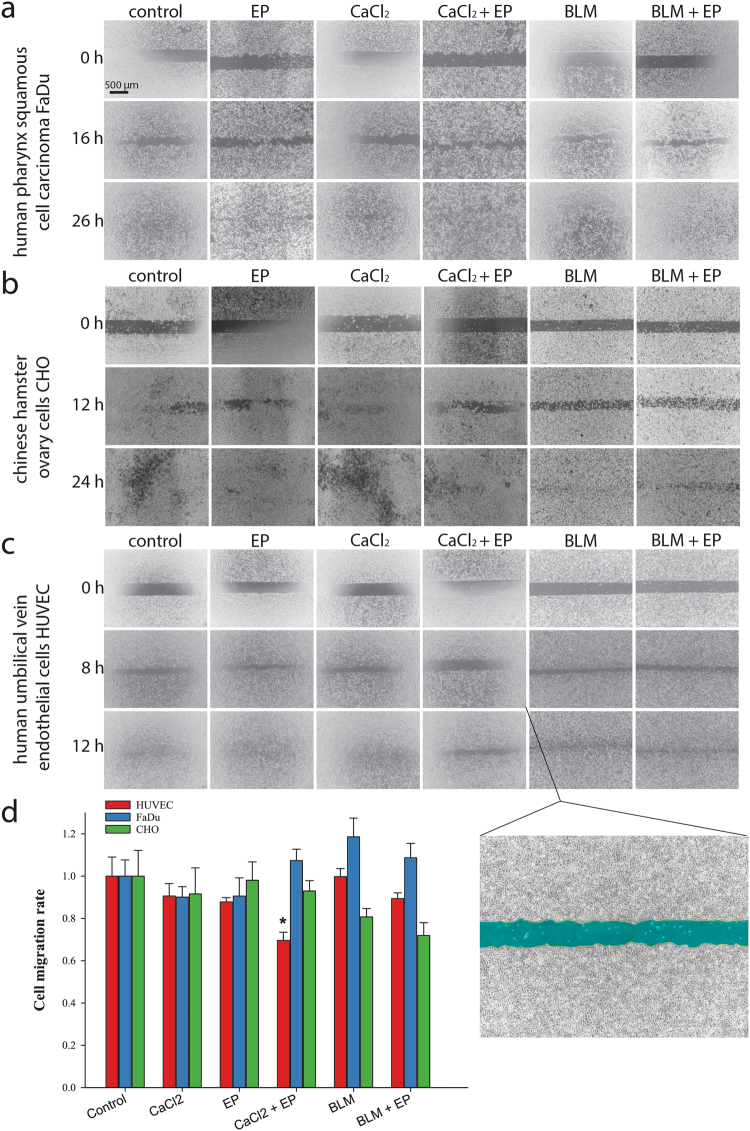
Reduced migration potential of HUVEC endothelial cells after CaEP or ECT with bleomycin (BLM). (**a**–**c**) Original images of wound healing assays taken at different time points after removal of silicone inserts. Last shown time point is when experiments were terminated (when controls have sealed). (**d**) The kinetic analysis of cell migration rate (n = 4 wells in at least 2 independent experiments). Bars represent AM ± s.e.m. Asterisk indicates statistically significant difference (*p < 0.05) compared to control, performed by Holm-Sidak testing following one-way ANOVA. All data are normalized to the untreated cells. Magnified image shows an example of the cell-free area (artificially coloured blue), that was quantified at each time point.

To determine the anti-vascular potential, an endothelial cell tube formation assay was performed on HUVECs. The ability of the cells to form capillary-like structures (Fig. 3) after CaEP was significantly reduced, as cells were unable to attach to the underlying matrix and form the tubes for at least 6 hours after treatment. ECT with bleomycin had no significant effect on tube formation. To determine effects of CaEP on other biological properties, such as cell migration, a wound healing assay was performed (Fig. 4). Statistically significant inhibition of cell migration after CaEP was observed specifically in 20–40% of HUVECs (Fig. 4d), whereas ECT with bleomycin had no such effect in any of the tested cell lines.

### CaEP has an anti-vascular as well as an anti-tumour effect *in vivo*

**Figure 5:**
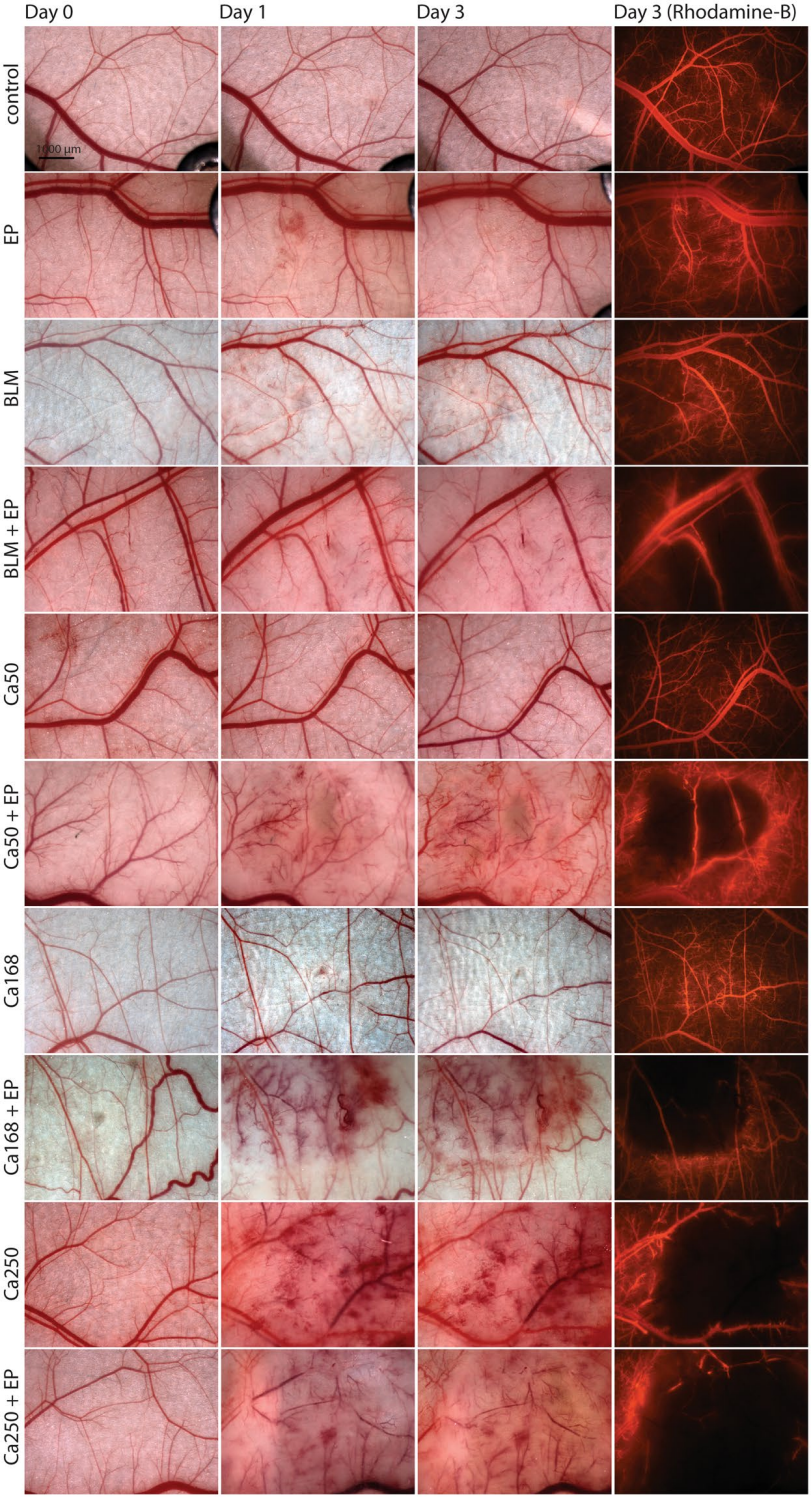
Damage of normal blood vessels in dorsal window chamber after CaEP or ECT with bleomycin (BLM). Bright-field images of vasculature in dorsal window chamber before the therapy (Day 0) and after the therapy (Day 1 and 3) and images of fluorescent blood vessels (Rhodamine B fluorescence). For each group, two to three mice were randomly assigned.

**Figure 6:**
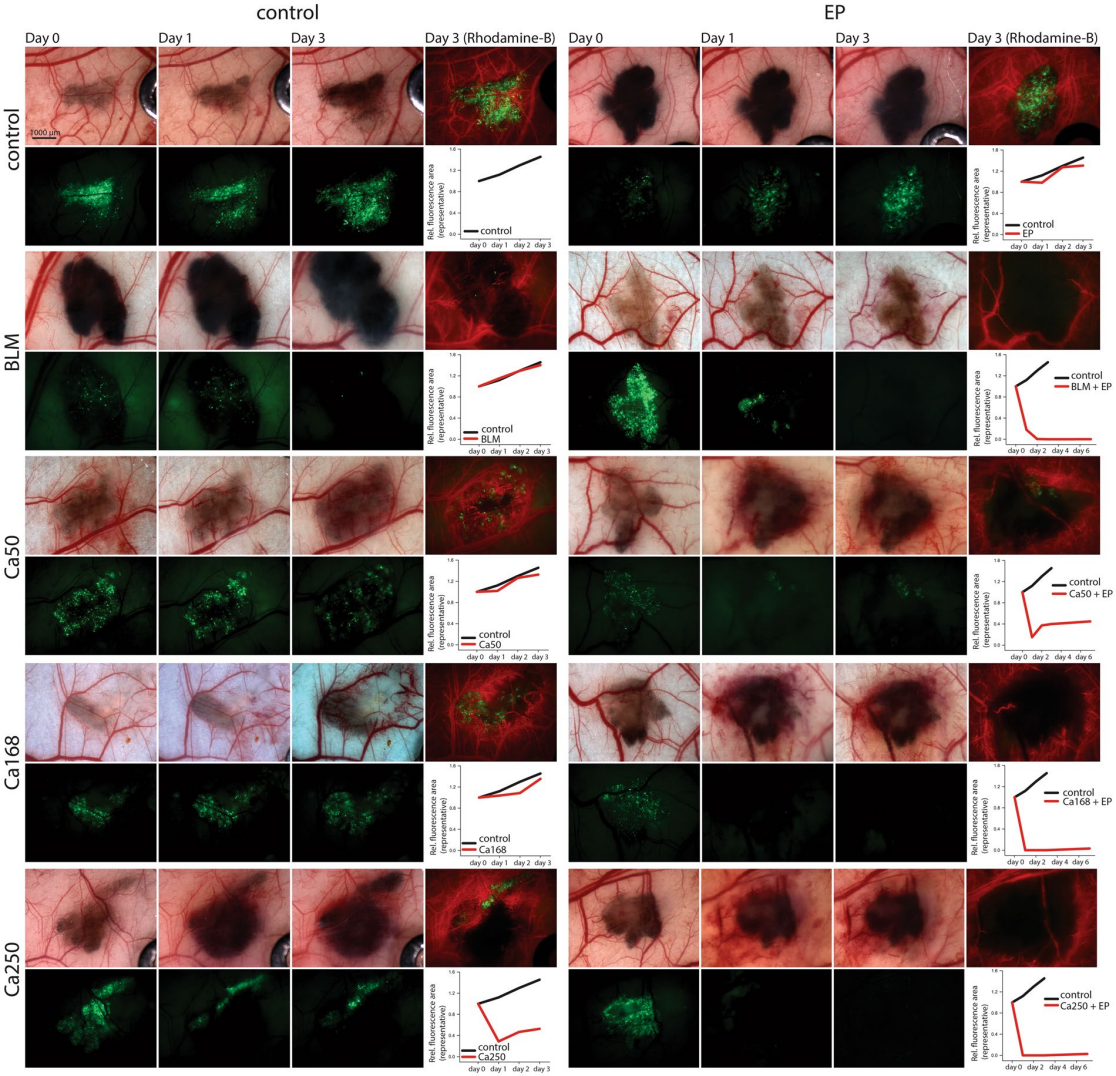
Damage of tumour blood vessels and tumours in dorsal window chamber after CaEP or ECT with bleomycin (BLM). Bright-field images of tumours and blood vessels in dorsal window chamber before the therapy (Day 0) and fluorescence images of tumours (green) and blood vessels (red) on day 1 and 3 after the therapy. Graphs demonstrate fluorescence area of representative tumours indicating tumour growth normalized to day 0 (control – black line; treated tumour – red line). For each group, two to three mice were randomly assigned. Due to high melanin content and fast tumour growth, control tumours were monitored for only 3 days. Only best-responding tumours treated with combined therapy were monitored for up to 7 days (data not shown).

The *in vivo* anti-vascular effects of CaEP were evaluated by intravital microscopy in the dorsal window chamber model in normal and tumour blood vessels. Blood vessels were visualized with rhodamine-B labelled dextran on day 3 after treatment to determine vessel functionality. CaEP disrupted both normal (Fig. 5) and tumour blood vessels (Fig. 6) and caused tissue damage, similar to ECT with bleomycin. In all cases, larger vessels were damaged, while their functionality was preserved, whereas all smaller vessels in the treated area were destroyed. After 250 mM calcium, blood vessels at the injection site were damaged, even without electroporation. Calcium concentrations of 168 mM and 50 mM without EP did not affect the functionality of blood vessels. When calcium was combined with electroporation, this effect was amplified. The observed effects did not differ between normal and tumour vasculature.

Anti-tumour effects were also observed in B16F1 GFP melanoma tumours (Fig. 6). The effect on tumour survival was estimated based on loss of fluorescent signal, and it was followed up to 7 days after therapy in groups with combination therapies. Treatment with CaEP of tumours caused almost immediate necrosis, whereas ECT with bleomycin caused gradual death of tumour cells. The change in the appearance of a tumour, mainly presented as extravasation was already seen 1 h after the treatment (data not shown). In the tumours treated with CaEP and ECT, fluorescence was detected again on day 7, indicating that tumours started to re-grow.

**Figure 7:**
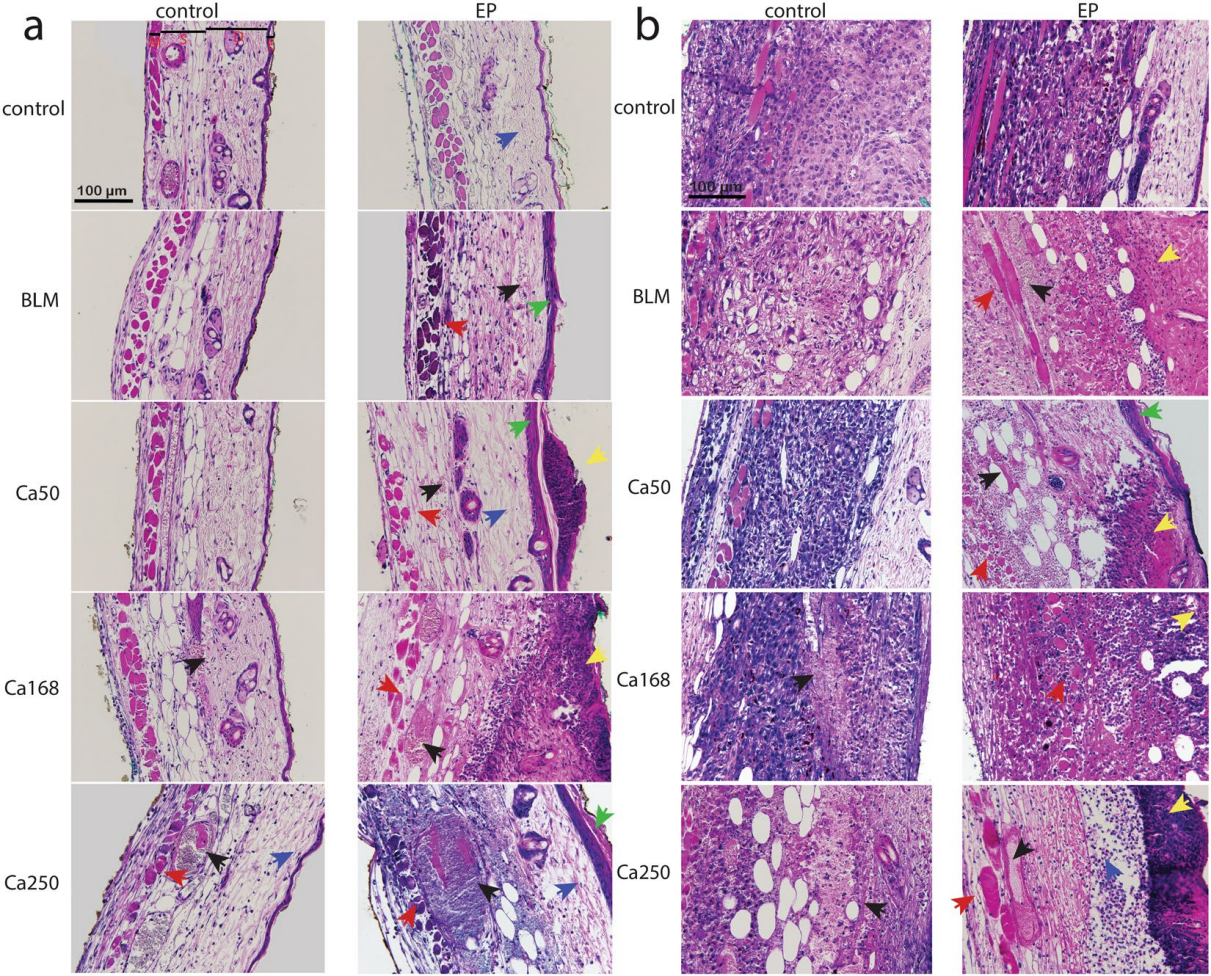
Tissue damage in DWC after CaEP or ECT with bleomycin (BLM). Samples of two mice per treated group were subjected to histological determination of tissue damage. (**a**) Representative images of normal tissue sections that were exposed to the treatment and stained with haematoxylin and eosin. *E* epidermis, *D* dermis with hair follicles, *S* subcutaneous tissues with blood vessels, *M* striated muscle layer. (**b**) Representative images of tumour sections exposed to the treatment. *Blue arrow* – oedema; *black arrow* – blood vessels and extravasation; *green arrow* – damaged epidermis; *red arrow* – muscle necrosis; *yellow arrow* – tissue necrosis.

Histological evaluation was performed 3 days after the treatment. In normal skin (Fig. 7a), calcium alone caused minimal extravasation of erythrocytes at 168 mM calcium. This was more pronounced at 250 mM, where necrosis of endothelial and muscle cells was also visible. Electroporation alone caused minor swelling (oedema) of the tissue. Necrosis of epidermal, endothelial and some muscle cells was observed after CaEP at 50 mM calcium. These features were more evident at higher calcium concentrations with scab formation. At 250 mM calcium, a typical blue staining of erythrocytes also occurred, showing calcium deposition in tissue. Bleomycin alone did not damage the normal tissue, while necrosis of epidermal, endothelial and muscle cells, was observed after ECT with bleomycin.

In tumours (Fig. 7b), only the highest concentration of calcium alone (250 mM) resulted in tumour cell necrosis, while lower concentrations of calcium and bleomycin alone did not affect the tumour cells. After CaEP and ECT with bleomycin, tumour cell necrosis was evident. At day 3 after treatment, progressive necrotic changes were observed in the tumours treated with CaEP, which became more evident with increasing calcium concentration, resulting in pyknotic cell nuclei and formation of a scab. In tumours treated with ECT with bleomycin, tumour cells were dying from both necrosis and apoptosis.

## Discussion

In this study, we demonstrated that CaEP has an anti-vascular effect on both normal and tumour blood vessels. CaEP inhibited the formation of capillary-like structures *in vitro*, and damaged tumour, as well as normal blood vessels, in a similar way as that of ECT with bleomycin. Larger vessels, arterioles and venules were less affected, and their functionality was preserved.

The anti-tumour effectiveness of CaEP has already been demonstrated *in vitro*, *in vivo* and in clinical studies^11,17,19,20,25^ but its effect on vasculature has not yet been explored. The doses of calcium used have been 1–5 mM CaCl_2_ for *in vitro* studies, where sensitivity is very high across different cell lines^11,15^. For *in vivo* studies, 168 mM has been used^11,18,25^, and recently, a broader range of concentrations have been explored *in vivo*^17^, in which a wide range of calcium doses was shown to be effective. In a clinical study on cutaneous tumours^19^, a dose of 220 mM (directly injected into the tumour in a volume equivalent to 50% of the tumour volume) was used in order to compensate for loss to the general circulation. In the present study, a range from 50 to 250 mM of calcium chloride has been investigated *in vivo*; for bleomycin, the standard dose^26^ has been used. Additionally, to further evaluate CaEP, we have chosen 4 different cell lines of varying tissue origin: B16F1 murine melanoma, FaDu human pharynx squamous cell carcinoma, CHO cells, and HUVECs. HUVECs were chosen for studying vascular effects, B16F1 cells were later on chosen for tumour model in *in vivo* study. Additionally, the CHO cell line was chosen, because it is a common research model in basic electroporation studies. In clinical practice, ECT is often used for treatment of head and neck cancers, therefore, to evaluate whether CaEP would be also applicable for this tumour type, we included in the study the FaDu (squamous cell carcinoma) cell line, which is one of the most used research models for head and neck tumours. Electroporation with increasing concentrations of calcium significantly reduced cell viability in a dose-dependent manner in all cell lines; however, their sensitivity to CaEP differed. The least sensitive were normal CHO cells and the most sensitive were B16F1 melanoma cells. The observed difference in sensitivity is in line with previous reports on CaEP toxicity^11,14^. It was demonstrated that normal cells have higher expression of calcium pumps, allowing them to remove excess of calcium from the cytosol faster than malignant cells^17,27^, which might be the reason why normal CHO cells were the least sensitive to CaEP. However, further studies are needed to explore the underlying mechanisms of different cell sensitivity to CaEP. Loss of ATP after CaEP was previously demonstrated where intracellular levels of ATP were measured on attached cells with the fluorescent dye quinacrine, and a significant decrease in fluorescence was detected at 15 minutes post-treatment^15^. Similar results have been obtained with different methods in other studies^11,18^. These studies support the hypothesis that cell death induced by CaEP could be a consequence of sudden ATP loss and severe impairment of ATP production; however, ATP depletion can also be a result of the certain type of cell death, so further studies are needed to explore the causation of observed ATP depletion. In contrast, ECT with bleomycin also caused dose-dependent decreases in cell viability, but ATP depletion was evident only at the highest doses, where most of the cells were dead. In both viability and ATP graphs, the results were normalized to untreated or EP-treated groups; therefore, the effect of EP alone is not shown. EP alone reduced cell survival (~25%) and caused some loss of ATP (~10%) in all cell lines. Therefore, the presented results only indicate the effect of the calcium and bleomycin that entered the cells.

According to previous studies, calcium overload can induce both apoptosis and necrosis. Cells committed to apoptosis but lacking ATP and sufficient clearance by immune cells will ultimately die by (secondary) necrosis^12,28,29^. Excessive uptake of calcium in the mitochondria is known to cause the formation of pores in the mitochondrial inner membrane, causing a mitochondrial permeability transition pores that can have destructive consequences because the mitochondrial inner membrane potential is disrupted. This stops the synthesis of ATP, which can compromise cellular homeostasis and lead to cell death by necrosis^12^. It needs to be pointed out, that this is one of the possible scenarios for cell death; in reality, it is probably a mixture of different activated pathways since calcium is involved in a broad range of different cellular processes^6,30^. Additionally, in the studies on calcium overload induced by nanosecond pulsed electric fields, it was shown that calcium is involved in stabilization of pores in the cell membrane. This state was then replaced by sudden pore dilation and/or opening of new larger pores and the following cell death was not a consequence of osmotic swelling but rather because of membrane rupture. Also, calcium influx induced by nanosecond pulsed electric fields resulted in necrosis as a preferential mode of cell death^31,32^. These findings might be also true for conventional electroporation using microsecond pulses. The type of cell death in our study was determined by flow cytometric analysis of the cells stained with 7-AAD/FITC Annexin V and morphological changes of the cells. After CaEP, a high increase of cells in necrotic or in late necrotic/apoptotic phase i.e. secondary necrosis was seen when compared to control groups, which was expected based on the observed ATP depletion and morphological changes. In addition, *in vivo* studies show extensive tumour necrosis in histologically different types of tumour^11^. In contrast to CaEP, no increase in apoptotic and/or necrotic cells after ECT with bleomycin was seen when compared to control groups. Bleomycin causes DNA damage by making single- and double-strand breaks, and it induces two types of cell death: pseudoapoptosis or mitotic cell death, depending on the number of internalized bleomycin molecules^33,34^. However, in another study using mouse fibrosarcoma (WEHI-164) and normal rat skeletal muscle (L6) cell lines, apoptotic cells were also observed at 24 h after CaEP, as determined by Tunel assay^35^ and recently confirmed by ultrastructure analysis^27^.

One of the hallmarks of the metastatic process is the ability of cells to migrate^36^. To evaluate whether CaEP has an effect on this process, the effect of CaEP on the migration of cells that survived the treatment was investigated. Cell migration rates were evaluated with an *in vitro* wound healing assay. The migration rate of tumour cells was not affected after CaEP, similarly was previously shown for ECT with either bleomycin or cisplatin in melanoma cells^37^. However, our data demonstrated inhibition in the migration of HUVECs after CaEP. ECT with bleomycin did not have a statistically significant effect on the migratory potential of any of the cell lines. Furthermore, the ability of HUVECs to form capillary-like structures *in vitro* was tested after CaEP and ECT with bleomycin. The results showed that capillary-like tube formation was inhibited after CaEP, but not after ECT. We also observed that cells did not attach to the matrix for several hours after CaEP treatment. The mechanism behind this phenomenon could be the depolymerisation of the actin cytoskeleton due to lower intracellular ATP concentrations^38^. It is a well-known fact that the highly dynamic process of cell migration is driven by focal adhesion turnover and actin cytoskeleton remodelling. For proper focal adhesion dynamics, the integrity of the mitochondria must be maintained, since its physiology has an important role in cell migration and cytoskeleton dynamics^38,39^. In addition, actin is also regulated by calcium. Increased levels of calcium lead to fragmentation of F-actin fibres, disruption of the actin structure, and detachment of the cell cortex from the cell membrane. Increased calcium concentrations destabilize microtubules through calmodulin and activate calpains, which cleave microtubules^12,13^. Additionally, leakage of ROS as a consequence of mitochondrial impairment can occur^40^. All these factors can disturb actin cytoskeleton polymerisation, leading to the observed inhibition of cell migration and tube formation of endothelial cells^12^. ECT with bleomycin had no significant effect on cell migration and tube formation; although it is known that it has an anti-vascular effect *in vivo*. The reason for this is likely the short observation time (up to 6 hours), as bleomycin-induced cell death mainly becomes evident when cells divide^33^. After CaEP, this effect is immediate.

**Figure 8:**
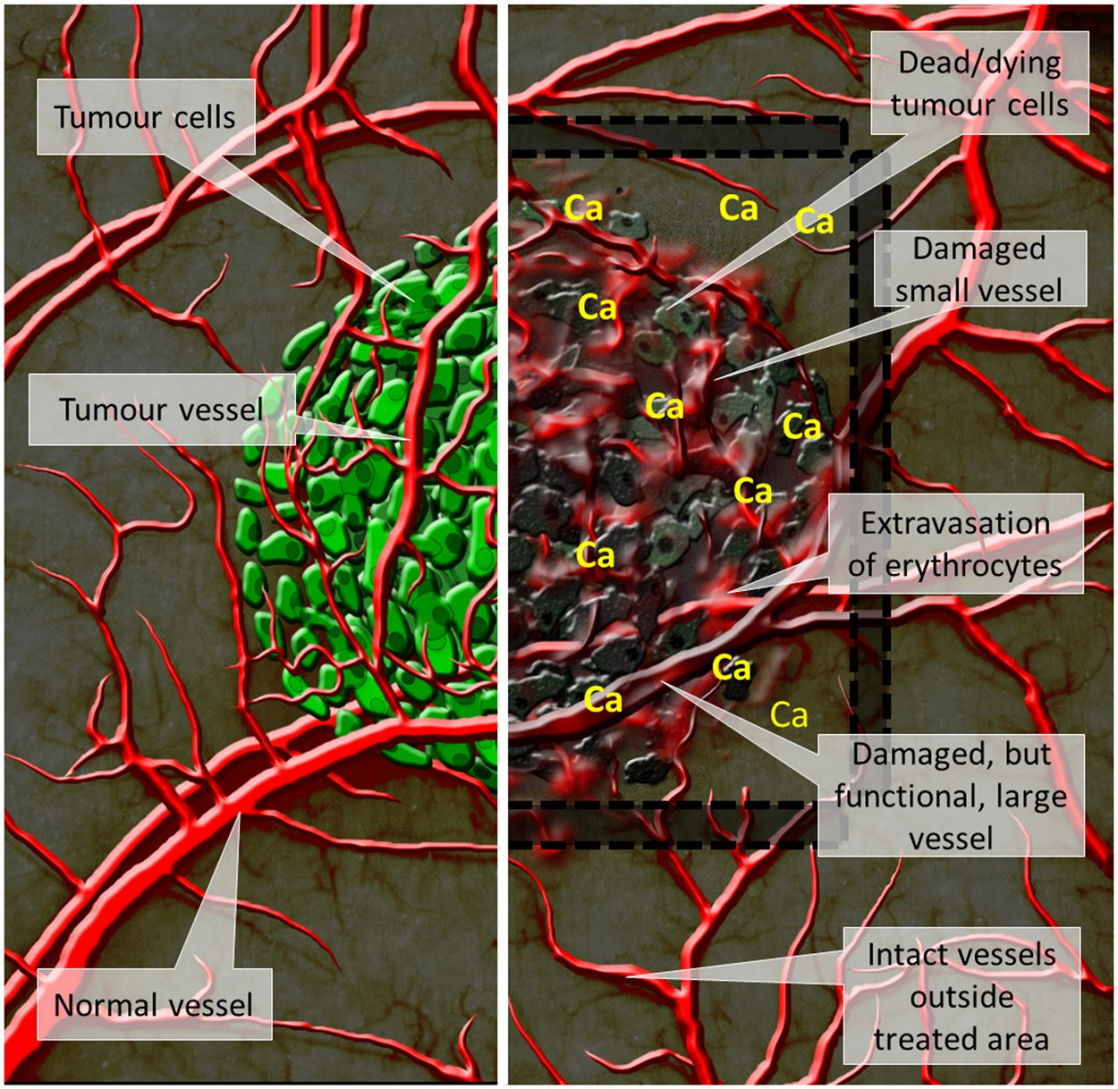
Scheme of cytotoxic and vascular effects after CaEP. The left side of the figure represents intact normal and tumour vessels and viable tumour cells. On the right side positions of electrodes (marked with black lines and shaded area) and effects after CaEP in the treated area are shown, such as dead or dying tumour cells, damage to the small blood vessels, which results in extravasation of erythrocytes, damaged but functional larger vessels and intact vessels outside of the treated area.

The *in vitro* results from our study indicated an anti-vascular potential for CaEP; thus, we further investigated the effect of CaEP on normal and tumour vasculature in a dorsal window chamber model. Three different calcium concentrations in combination with electroporation were compared to ECT with bleomycin, which has a proven vascular disrupting action^21,22^. Lower concentrations of calcium (50 mM and 168 mM) without electroporation caused minor blood extravasation; no other damage was observed in normal blood vessels. In contrast, the high dose of 250 mM calcium damaged vessels at the injection site. Electroporation potentiates these effects by causing severe normal blood vessel and tissue damage (tissue necrosis). ECT with bleomycin affected smaller vessels, leaving larger vessels intact and functional, which has been demonstrated in other studies^22,41^. Similarly, the functionality of some larger vessels was preserved after CaEP as well. The effect of both CaEP and ECT with bleomycin on tumour blood vessels was the same as on normal vessels in this model, thus CaEP and ECT with bleomycin did not exhibit any selectivity to tumour blood vessels. However, due to the small size of the tumours in the dorsal window chamber and the corresponding tumour vasculature consisting of smaller vessels, all tumour blood vessels were destroyed, and only larger, normal blood vessels remained in the treated area (Fig. 8). Similar changes in tumours that were viewed in a dorsal window chamber were previously described for ECT with bleomycin in a human colon carcinoma tumour model growing in SCID mice^22^.

When comparing the effect of calcium injection alone *versus* CaEP on tumour growth, our results indicated that calcium alone had no evident effect on tumour growth of B16F1 melanoma, with the exception of the 250 mM calcium concentration, which reduced tumour growth. CaEP induced an immediately visible change in a tumour; the area of a tumour became darker (extravasation), which was observed while monitoring the animals after the therapy (data not shown). A tumour disappeared completely within one day, and the time for regrowth of tumours depended on the calcium concentration; the lowest calcium concentration (50 mM) caused only short growth delay, while regrowth occurred after 7 days with the higher concentrations. ECT with bleomycin caused gradual death of tumour cells, rather than immediate death as for CaEP, and was accompanied by local tissue oedema. The tumours completely disappeared after 3 days; however, tumours started to re-grow after 7 days in all cases, which might be due to the survival of some tumour cells after CaEP and/or to the preservation of blood vessels functionality, thus providing the oxygen and nutrients for the survived tumour cells. In other preclinical *in vivo* studies, the effect of CaEP was determined for tumours growing subcutaneously. Many histologically different types of tumours were tested, and the sensitivity between the tumour types was shown^17^. According to the study, lung carcinoma HT69 was the most responsive, while bladder carcinoma SW780 was the least responsive^17^. As evidenced by the level of necrosis that was observed after 168 mM CaEP in our study, melanoma tumours are very sensitive to CaEP. This was also demonstrated in a clinical study^19,20^. A patient with subcutaneous melanoma metastases was first treated with ECT, and then other nodules were treated with CaEP. The treated nodules disappeared after both treatments; however, after treatment with CaEP, a strong immune response against melanoma cells occurred that also affected non-treated tumours^20^.

In conclusion, our study is the first to show that CaEP, similar to ECT with bleomycin, has an anti-vascular effect on both normal and tumour blood vessels. CaEP inhibited the formation of capillary-like structures *in vitro* and damaged normal and tumour blood vessels in the B16F1 melanoma tumour model *in vivo*. These results show that CaEP has a significant tumour necrosis effect. Our study provides further evidence for the use of CaEP in clinical settings.

## Methods

### Cell lines

B16F1 murine melanoma cells (ATCC) were cultured in Advanced MEM (Thermo Fisher Scientific), CHO cells (ATCC) were cultured in Advanced DMEM/F-12 (Thermo Fisher Scientific), HUVECs (ATCC) were cultured in Advanced DMEM (Thermo Fisher Scientific), FaDu human pharynx squamous cell carcinoma (ATCC) were cultured in Advanced RPMI 1640 (Thermo Fisher Scientific). All culture media were supplemented with 5% foetal bovine serum (FBS), 10 mM L-glutamine (Thermo Fisher Scientific), 50 mg/ml gentamicin and 100 U/ml penicillin and held in a 5% CO_2_ humidified incubator at 37 °C.

B16F1 cell line expressing the green fluorescent protein (B16F1 GFP) was prepared by stable transfection with pEGFP-N1 plasmid (Invivogen) using electroporation as previously described^42^ and cultivated with the addition of 2 mg/ml of selection antibiotic geneticin (Thermo Fisher Scientific).

### Animals

Eight to twelve-week-old female C57BL/6JOlaHsd mice weighing 18–21 g were used in experiments. Mice were kept in quarantine for 2 weeks before the experiment. Specific-pathogen-free housing conditions were provided throughout the period of quarantine and experiment. The experimental procedures were performed in compliance with the guidelines for animal experiments of the EU directive (2010/63/EU) and the permission from the Veterinary Administration of the Ministry of Agriculture, Forestry and Food of the Republic of Slovenia (permission no. U34401–1/2015/16). In the experiments, 2 mice were randomly assigned to an experimental group with single treatments (control, EP, BLM, Ca) and 3 mice in the combination groups (BLM + EP and CaEP).

### Drugs and solutions

For *in vitro* experiments, bleomycin (Bleomycin medac, Medac) was diluted in distilled water to concentrations of 1.4 × 10^−6^ M, 1.4 × 10^−7^ M, 1.4 × 10^−8^ M, 1.4 × 10^−9^ M or 1.4 × 10^−10^ M in a final cell suspension. Calcium solution was prepared from CaCl_2_ × 6H_2_O in distilled water to 250 mM in the stock solution. It was diluted to 5 × 10^−3 ^M, 4 × 10^−3 ^M, 3 × 10^−3 ^M, 2 × 10^−3 ^M or 1 × 10^−3 ^M in a final cell suspension. The electrical conductivity (measured with SevenCompact S230 conductometer (Mettler Toledo, Switzerland) at 24 °C) and the osmolality (measured by freezing point depression with Knauer cryoscopic unit (model 7312400000, Knauer, Germany)) of final solutions changed from 0.2 S/m to 0.3 S/m and from 0.16 Osm/kg to 0.19 Osm/kg, respectively. These changes had no influence on the outcome of electroporation^43,44^.

For *in vivo* experiments, 250 × 10^−3^ M, 168 × 10^−3 ^M and 50 × 10^−3 ^M calcium solutions and 1.4 × 10^−3^ M bleomycin solution diluted in distilled water were used.

### Electroporation protocol

Cells were collected by trypsinization and centrifugation, washed in ice-cold electroporation buffer (125 mM sucrose, 10 mM K_2_HPO_4_, 2.5 mM KH_2_PO_4_, 2 mM MgCl_2_ × 6 H_2_O) and prepared for electroporation as previously described^45^. Briefly, 50 μl of the suspension (1 × 10^6^ cells) served as a control for treatment, while an additional 50 μl was pipetted between two stainless-steel parallel-plate custom-made electrodes (2 mM apart), and 8 square-wave electric pulses (1300 V/cm, 100 μs, 1 Hz) were generated with an electric pulse generator GT-01 (Faculty of Electrical Engineering, University of Ljubljana, Ljubljana, SI). Pulses were monitored on an oscilloscope Tektronix TDS3012 (Tektronix). After application of electric pulses, cells were transferred to a 24-well ultra-low attachment plate and incubated for 5 min at room temperature, then 1 ml of culture medium was added to the cells, and the cells were utilized in the different assays (see below).

### Cytotoxicity assay

To determine cell viability after treatment with calcium and bleomycin alone or in combination with EP, a Presto Blue (Thermo Fisher Scientific P#A13262) assay was performed as previously described^46^. Briefly, from the 1 ml cell suspension (see above), cells were diluted to 1 × 10^3^/100 µl (control groups) or 2 × 10^3^/100 µl (EP groups) and transferred to 16 wells per group in a 96-well Tissue culture plate (VWR), each well thus containing 1 × 10^3^ cells, when they were exposed to drug alone or 2 × 10^3^ cells exposed to combined treatment with EP. The plates were then incubated for 3 days. Relative survival rate values were normalized to EP group alone or untreated control group. IC_50_ concentrations (50% inhibition of survival) were obtained from the survival curves of cells exposed to combined treatments.

### ATP assay

To determine ATP loss after treatment, ATP levels in the cell lysates were measured. After treatment, 1 × 10^6^ cells were incubated in 1 ml of complete culture media for 1 h at 37 °C in a 5% CO_2_ atmosphere. Afterwards, cells were lysed with boiling distilled water as previously described^47^. ATP content was determined using an ATP determination kit (Molecular Probes P#A22066) according to manufacturer’s instructions, and bioluminescence was measured in white 96-well plates. Relative ATP content values were normalized to EP group alone or untreated control group.

### Giemsa staining

For morphological determination of cell death, cytospins were prepared. Cells (5 × 10^3^), treated with IC_50_ concentrations of the drug alone or in combination with EP, were added to a slide chamber and centrifuged for 4 minutes at 1,000 rpm in a cytocentrifuge. The slides were air dried, fixed in absolute methanol (approximately 1 h after treatment) and stained with Giemsa’s Azure methylene blue solution (Merck). Images of the cells were captured with a DP72 CCD camera connected to Olympus BX-51 microscope.

### Determination of cell death mechanisms

Cell death mechanisms were determined with a FITC Annexin V Apoptosis Detection Kit with 7/AAD (Biolegend P#640922) according to manufacturer’s instructions. Briefly, after 5 minutes post-treatment incubation time, cells were transferred to the 15 ml tubes and washed twice by centrifugation. Then, cells were resuspended in Annexin V Binding Buffer and transferred to flow cytometry tubes. FITC Annexin V and 7/AAD were added to the cells 40–45 minutes after electroporation and incubated for another 15 minutes prior measurement. The measurements were performed using FACSCanto II flow cytometer (BD Biosciences). A 488-nm laser was used for the excitation; both 530 and 650-nm band-pass filters were used for detection of green and red fluorescence. For each sample, at least 100000 events were measured. The data were analysed using BD FACSDiva software version 8.0.1 (BD Biosciences). All debris was excluded for further analysis using FSC/SSC scatter plot.

### Tube formation assay

To determine the effect of CaEP and ECT on the ability of endothelial cells to form capillary-like structures, a tube formation assay was performed on HUVECs. No treatment control, EP control, and cells treated with IC_50_ concentrations (with or without EP) were plated in a density of 2.4 × 10^4^ cells per well on a µ-Slide Angiogenesis (Ibidi) covered with a Matrigel® Basement Membrane Matrix Phenol Red-Free (Corning) and incubated for 4 hours until the formation of tubular complexes. The cells were then stained with Calcein-AM (Merck) and their images were captured with a DP72 CCD camera connected to Olympus IX-70 inverted fluorescence microscope. Cellular networks were analysed using Angiogenesis Analyzer for ImageJ^48^ by quantifying a number of nodes, junctions and meshes, total branching length and total segments length.

### Wound healing assay *in vitro*

To determine the migratory capacity of the treated cells, wound healing assays were performed^49^ after treatment with IC_50_ concentrations alone with or without EP. After treatment, 4 × 10^4^ cells were plated on a 24-well plate with a silicone insert (Ibidi) that formed a 500-μm ± 50 μm cell-free gap after removal. Cells were incubated overnight at 5% CO_2_ and 37 °C until a confluent cell monolayer was formed, then the silicone inserts were removed. Images of the wounds were captured with DP72 CCD camera connected to Olympus IX-70 inverted microscope at 0 h (when culture inserts were removed) and then every 2–4 hours (depending on the cell line used and optimized in preliminary tests), until the wounds of the control group were completely sealed (for HUVEC 12 h, CHO 24 h, FaDu 26 h). At each time point, the cell-free area was quantified using FIJI image analysis software^50^ and a kinetic analysis was made from the obtained values. The cell migration rate of each experimental group was normalized to the migration rate of untreated cells. The assay was not performed in B16F1 cells because these cells have very limited migratory potential.

### Dorsal window chamber

For implantation of dorsal window chambers, mice were anaesthetized with an anaesthetic solution (ketamine, 125 mg/kg; xylazine 12.5 mg/kg; acepromazine 2.5 mg/kg) injected intraperitoneally with volumes adjusted to the weight of the animal. The back of the mouse was shaved and depilated with a depilatory cream. Mice were kept warm using heating pads throughout the procedure. The dorsal window chamber was implanted surgically onto the extended double layer of skin as previously described^51^. After surgery and on the following two days, 50 µl of analgesia (ketoprofen, 3.5 mg/kg) was injected intramuscularly. In the experiments investigating the effect of CaEP on tumour vasculature, 2 days after surgery tumours were induced by injection of 3.5 × 10^5^ B16F1 GFP cells in 5 µl of physiological solution into the dermis of intact skin in the dorsal window chamber with a 29-G needle.

### *In vivo* electroporation and intravital imaging

For intravital imaging, a Carl Zeiss SteREO Lumar V12 fluorescence stereomicroscope equipped with a Neo-Lumar S 0.8x objective and an AxioCam MRc5 digital camera was used, and effects on tumour and normal vasculature were observed. Mice were anaesthetized with isoflurane inhalation anaesthesia and the dorsal window chamber was fixed on a custom-designed holder. Five days after implantation (when tumours reached the size of 4–5 mm^3^), dorsal window chambers were imaged followed by treatment (Day 0). Either 5 µl of MiliQ water or different concentrations of calcium or bleomycin (see above) were injected intratumourally or intradermally in the dorsal window chamber region. Electrodes were placed around the injection site, and good contact between the skin and the electrodes was ensured by using conductive gel. EP was performed immediately after injection by application of 8 (4 + 4) square-wave electric pulses given in perpendicular directions through parallel electrodes with an inner distance of 4 mm. Pulsing conditions were 1300 V/cm, 100 µs and 1 Hz. Pulses were generated by ELECTRO cell B10 HVLV (LEROY biotech, Betatech, Saint-Orens-de-Gameville, France). Dorsal window chambers were imaged once per day for 3 days. On day 3, the functionality of normal and tumour blood vessels was visualized with the injection of Rhodamine B-labelled dextran (70 kDa; Merck) into the orbital plexus and images of fluorescent blood vessels were taken. Tumours treated with a combination of electroporation and calcium or bleomycin were additionally imaged on day 7. The fluorescence area was measured for each tumour using FIJI software^50^.

### Histological determination of skin damage

For the histological determination of the skin damage after therapy, paraffin-embedded sections were prepared. Mice were sacrificed by cervical dislocation at day 3, and the region of skin in the dorsal window chamber was excised. The excised skin was then fixed in zinc fixative for 1 day and then stored in 70% ethanol until it was embedded in paraffin for histological observation of skin damage. In the direction perpendicular to the skin layers, 5-µm thick sections were cut and stained with haematoxylin and eosin. Images were taken with Olympus BX-51 microscope equipped with a digital camera DP72.
